# Responder perspectives on preparedness for healthcare needs of vulnerable populations during floods and heatwaves: a qualitative study in Emilia-Romagna, Italy

**DOI:** 10.1136/bmjph-2024-002459

**Published:** 2025-09-12

**Authors:** Giorgia Soldà, Helle Molsted-Alvesson, Marco Montalti, Chiara Reali, Davide Gori, Johan Von Schreeb, Petter Ljungman

**Affiliations:** 1 Institute of Environmental Medicine, Karolinska Institute, Stockholm, Sweden; 2 Department of Global Public Health, Karolinska Institutet, Stockholm, Stockholm County, Sweden; 3 Dipartimento di Scienze Mediche e Chirurgiche, University of Bologna, Bologna, Italy; 4 Unit of Hygiene and Public Health Forlì-Cesena, Department of Public Health, Romagna Local Health Authority, Ravenna, Italy; 5 Department of Global Public Health, Center for Research on Health Care in Disasters, Karolinska Institute, Stockholm, Sweden; 6 Department of Cardiology, Danderyd University Hospital, Stockholm, Sweden

**Keywords:** Health Services Accessibility, Cross-Sectional Studies, Emergencies, Sociodemographic Factors, Qualitative Research

## Abstract

**Introduction:**

Climate change poses significant challenges to public health, exacerbated by the increasing frequency and intensity of extreme weather events. Emilia-Romagna, Italy, has recently experienced severe extreme weather events, including heatwaves and floods, prompting a need to assess the healthcare system’s capacity to respond to such events. Despite national initiatives, there is limited understanding of how vulnerable populations’ healthcare needs are addressed during climate-induced crises. This study explored preparedness and response strategies adopted by healthcare responders and close civil protection system stakeholders in addressing the healthcare needs of vulnerable populations during extreme weather events, such as floods and heatwaves, in Emilia-Romagna.

**Methods:**

We conducted a qualitative study in Emilia-Romagna’s Forlì-Cesena province, involving 10 key informants from the health district and related disaster management institutions within the civil protection system. We investigated preparedness, response and adaptation strategies for heatwaves and floods. Semistructured interviews were conducted, recorded and then transcribed. We employed reflexive thematic analysis, guided by the preparedness cycle framework.

**Results:**

An overarching theme was identified: ‘Flexible collaboration: adapting together in crisis’ comprising three subthemes: enhancing adaptability through teamwork, balancing flexibility and consistency in response and rethinking vulnerabilities and risk perceptions. Interviews indicated a lack of experience in activating heatwave early warning systems within civil protection plans. However, the frequent health emergencies, including COVID-19, floods and an earthquake, provided a form of ‘real-life training’ that enhanced response coordination and functionality during crises.

**Conclusion:**

The findings suggest the importance of flexibility and multilevel collaboration across the healthcare sector and its stakeholders in responding to climate-induced crises, such as heatwaves and floods. While coordination was strong during the response phase, the results suggest a lack of efforts in integrating community engagement in long-term recovery and adaptation planning.

**WHAT IS ALREADY KNOWN ON THIS TOPIC**

- Climate change increases the frequency and intensity of extreme weather events, placing significant pressure on healthcare systems. Traditional disaster preparedness frameworks often overlook the need for long-term adaptation in response to evolving climate impacts like floods and heatwaves.

**WHAT THIS STUDY ADDS**

- This study suggests the necessity of incorporating an adaptation phase into traditional disaster preparedness frameworks. It highlights the potential benefits of fostering flexible, multilevel collaboration among healthcare responders and civil protection stakeholders. Additionally, it indicates the importance of continuously updating definitions of vulnerability to better reflect the realities of climate-related hazards, as inferred from the perspectives of key informants.

**HOW THIS STUDY MIGHT AFFECT RESEARCH, PRACTICE, OR POLICY**

- The findings indicate that healthcare systems may benefit from adopting proactive strategies for managing climate crises. While the insights derived from this qualitative study provide a valuable foundation, further systematic research is essential to strengthen the understanding of effective practices.

## Introduction

 Climate change is a global phenomenon that poses significant threats to public health, through the increasing frequency and intensity of climate change-induced extreme weather events, such as floods and heatwaves.^1 2^ These hazards disproportionately affect vulnerable populations, including bedridden people, elders, socially isolated individuals, people with mental health conditions and individuals with cardiovascular, respiratory or renal diseases.^3^,^5^

Globally, climate-driven events have intensified: in 2022 alone, over 150 million additional people experienced food insecurity due to droughts and heatwaves.^1 2^ Rising risks of flooding, waterborne diseases and heat-related illnesses place mounting pressure on health systems and expose gaps in their preparedness and response.^1^

Emilia-Romagna, in northern Italy, has recently faced a series of climatic shocks.^6 7^

In May 2023, heavy floods and landslides caused 17 fatalities, 500 000 displaced people, 23 flooded rivers, 280 landslides and 400 blocked roads. As a consequence, the National Civil Protection Service was mobilised and the state of emergency—lasting 12 months—was declared.^8^,^10^

These events followed the COVID-19 pandemic and were compounded by an earthquake in September 2023 (3 km SW of Marradi, Tuscan-Romagna Apennines, ML 4.8, Mw 4.9) that occurred in the area.^11^

These events and relative public health emergencies have had a significant impact on the region’s environment, infrastructure, economy and the well-being of its residents. In addition, the region has witnessed an increase in the frequency and intensity of heatwaves, which can have significant health effects on vulnerable populations, including infants, the elderly and pregnant women.^12 13^

National response frameworks—such as the 2005 National Prevention Plan for Heatwave Health Consequences—have improved preparedness, especially by identifying at-risk populations and coordinating support at local levels, and have contributed to a decrease in heat-related deaths throughout the country.^13 14^ Reforms in the national civil protection service have led to a more systematic approach to disaster planning, including flood management, at different territorial levels, including Emilia-Romagna cities.^15^

Research on the health effects of flooding has revealed significant impacts, including increased incidence of infectious diseases, trauma, water-borne and vector-borne diseases and mental health issues such as anxiety and post-traumatic stress disorder.^16^,^22^ Flooding often exacerbates skin and respiratory conditions due to contaminated environments.

In the case of heatwaves, studies indicate a clear link to higher mortality rates among vulnerable populations, alongside heightened cardiovascular, renal and respiratory disorders, as well as mental health challenges.^23 24^

However, there is a notable gap in research regarding how health systems address the specific needs of vulnerable populations during climate shocks. Limited studies have explored the effectiveness of early warning systems, preparedness strategies and adaptation measures in Italy and Europe. Additionally, systemic approaches to health crisis management are underrepresented, constraining our understanding of effective responses to climate-related health threats.^25^

The aim of this qualitative study is to explore such gaps in the context of recent events of heatwaves and floods in the Emilia-Romagna region in Italy.

## Methods

To ensure comprehensive reporting, this paper follows the guidelines outlined in the Consolidated Criteria for Reporting Qualitative Research checklist.^26^

### Conceptual framework

Our study design departs from the preparedness cycle theoretical framework, traditionally applied in disaster management, incidents and health emergencies.^27^,^30^ This cycle emphasises a continuous approach to maintaining preparedness through a sequence of activities that can be summarised—although sometimes with different labels—in a sequence of these following steps: assessing, planning, training, exercising and revising plans.^27^,^30^ The core objective of this cycle is to maintain readiness and effectiveness in responding to recurrent and unexpected emergencies through regular exercises and updates to preparedness plans. While traditional disaster management frameworks often treat events like heatwaves and floods as isolated incidents, climate change demands a broader, more integrated approach. We therefore extend this cycle by incorporating a new step between recovery and anticipation, which we call ‘adaptation’. The adaptation step enhances resilience to future extreme weather events by systematically integrating insights from previous experiences into preparedness and response strategies, ensuring long-term sustainability in addressing the evolving nature of climate-related hazards.

**Figure 1:**
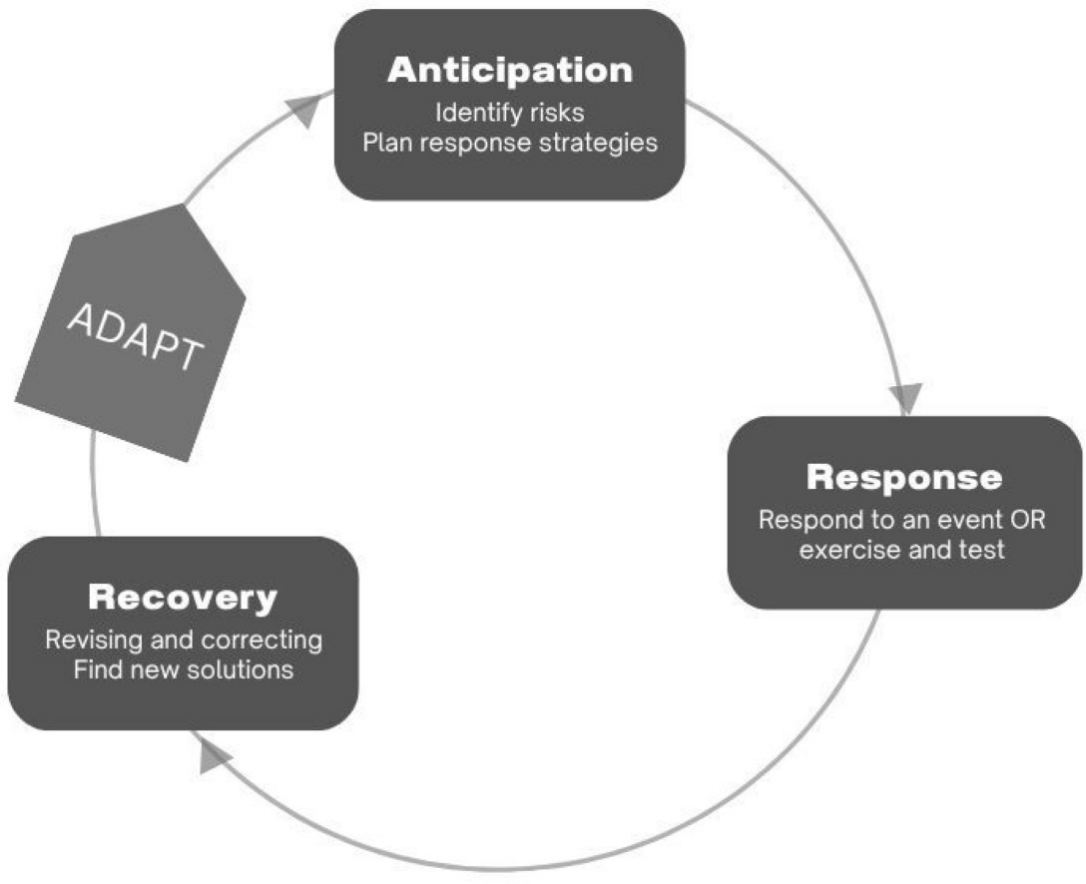
The preparedness cycle.

Our approach moves beyond the isolated disaster perspective, addressing the need to understand healthcare preparedness and adaptation strategies for vulnerable populations during extreme weather events, a need driven by the scientific community’s recognition of the escalating impacts of climate-driven events like heatwaves and floods^30^,^33^ (figure 1**,** (online supplemental figure 1).

### Study setting

The study was conducted in the province of Forlì-Cesena, part of Emilia-Romagna, Northern Italy, a region with a population of 4.46 million and a predominantly public health system. Our focus was on a population of 400 000 people, covered by three local health districts (Forlì, Cesena and Valle Savio, Rubicone) under the Romagna Local Health Authority.

Natural disaster management in this area falls under civil protection (including the prefecture and the mayor), while heatwave response is managed mainly by the healthcare system.

### Civil protection and heatwave plans

In Italy, the civil protection is a national service that includes the National Fire Brigade, armed forces, police forces, national research bodies and institutes and the Italian Red Cross, among others. The system relies on the principle of subsidiarity or integration. Subsidiarity refers to the assumption that decisions should be made at the most local level possible, unless a higher authority is better positioned to act. The first response to emergencies, regardless of their nature and extent, is thus guaranteed at the local level, starting from the municipal structure, the closest institution to the citizen, entrusting the intervention to the organisation closest to the territory in terms of the level of knowledge and competence.^15^

The National Prevention Plan for heat effects on health instead includes targeted interventions, such as identifying at-risk subgroups, issuing tailored heat health warnings, activating local support services like home visits and teleassistance for vulnerable individuals, and disseminating health recommendations during periods of elevated heat risk.

We conducted a document review of both the Intermunicipal Civil Protection Plans and the National Prevention Plan for heat effects on health, among others (online supplemental table 1).^934^,^37^ The national heatwave plan was notably detailed in identifying vulnerable groups and specifying roles during various alert levels, while local procedures were more concise and technical, reflecting their operational nature. In contrast, the civil protection plans emphasised coordination and territorial management during disasters like floods. The document review helped identify key informants and shaped our interview questions, allowing us to probe specific differences in how these plans are executed, ensuring a targeted investigation into their operational and strategic impacts.

The interview guide was structured around several key themes, including definitions of vulnerability in the context of floods and heatwaves, identification and prioritisation of vulnerable population groups, roles and responsibilities in preparedness and response planning, risk communication practices and intersectoral coordination. While the guide helped ensure consistency across interviews, the design allowed for flexibility and open-ended responses.

The interview guide with questions and probes (online supplemental material) was tested and refined through a pilot interview with a public health officer.

The concept of ‘vulnerable populations’ was addressed both through reference to national preparedness guidance (eg, heatwave and civil protection plans) and through targeted interview questions. In the interview guide, participants were invited to reflect on how vulnerability should be defined and assessed in their professional context, which population groups should be prioritised, what specific health needs should be considered and who holds responsibility for identifying and supporting vulnerable individuals before and during heatwaves and floods.

### Participant selection and recruitment

We purposively selected 11 key informants involved in preparedness and response for natural disasters within the health system and civil protection sectors in the Emilia-Romagna province object to the study (table 1). Guided by the concept of information power, we identified participants whose institutional roles gave them direct responsibility over key preparedness and response functions, such as managing alert levels, coordinating risk communication, identifying vulnerable populations and overseeing evacuation procedures.^38^ These roles were mapped using the local civil protection and heatwave plans to ensure alignment with the study aims. Only individuals with direct operational decision-making responsibilities were included, as they were uniquely positioned to provide insights into planning, coordination and intersectoral collaboration during recent heatwaves and floods. Because these were high-level institutional roles, each was typically occupied by a single individual within their respective organisation. Formal recruitment took place via email.

**Table 1:** Characteristics of the study sites and participants

Role	Source	Professional area (peacetime)	Professional area (crisis time)
Health district management	Local health districts 1, 2 and 3	Public and primary healthcare, healthcare management, healthcare services organisation	Health function within the municipality operations centre
Nursing services management	Local health district 1	Technical and nurse personnel and services coordination	Crisis unit coordinator
Psychological services management	Local health district 2	Psychotherapeutic support in the maternal and child ward	Crisis unit coordinator
Nursing services management	Local health district 2	Technical and nurse personnel and services coordination	Crisis unit coordinator
Primary Healthcare management	Local health districts 1, 2 and 3	Primary healthcare district services coordination, geriatrician	Crisis unit management
Social services officer	Local health district 2	Vulnerable individual healthcare—social setting transition support	Heatwave response coordinator
Civil protection managing officer	Civil protection	Training and support, coordination of volunteers	Support of the provincial emergency operations centre
Prefecture management	Prefecture	Prefecture administrative management, including public safety and security	Coordination of the provincial operating centre
Fire department management	Fire department	Fire brigades provincial level management	Support of the provincial emergency operations centre
Mayor office management	Municipality	Municipality management	Head of the municipality operations centre
Mayor office management	Municipality	Municipality management	Head of the municipality operations centre

Gender balance was respected (five female and five male participants). Interviews were arranged between July and August 2023, conducted in September 2023 and transcribed within the following 24 hours.

The study was approved by the Ethical Committee of the University of Bologna and written informed consent was obtained from all participants.

No patients or lay members of the public were involved in the design, conduct or reporting of this study. However, key informants were frontline public health and civil protection professionals, whose insights reflect community-level operational experience. A feedback meeting is planned to share the study results with participants and institutional stakeholders, supporting transparency and engagement.

### Data collection

Data were collected through inperson semistructured interviews conducted in Italian during September 2023 by GS.

Interviews were recorded and notes of non-verbal information and impressions during semi-structured interviews were supplemented after each interview. Transcripts were not returned to the participants for comments or collection. The average interview duration was 40 min.

### Data analysis

We employed reflexive data-driven thematic analysis, as outlined by Braun and Clarke, which is grounded in an interpretive qualitative paradigm.^39 40^ This method, commonly used in applied qualitative health research, respects and acknowledges the subjectivity of participants’ accounts while considering the reflective influence of researchers’ interpretations.^41^ Our study took a critical orientation to explore how the broader social context may have influenced the expressed meanings of individuals.^42^ Interview recordings were transcribed in Italian using Microsoft Word’s artificial intelligence transcription tool (Microsoft 365, 2021, Microsoft Corporation, Redmond, Washington, USA), with manual postprocessing to correct errors and enhance accuracy. The analysis included the interview transcripts. Audio recording transcriptions were then uploaded to MAXQDA V.24 (MAXQDA 2024, Consult. Sozialforschung GmbH, Berlin, Germany). The analysis was performed on the original Italian data and only codes, themes and quotes were translated eventually into English by GS. Thematic analysis was conducted inductively, meaning that codes were derived from the data itself rather than from pre-existing theoretical frameworks, enabling the emergence of new insights grounded in participant narratives. Coding was conducted by the lead author (GS) using the original Italian transcripts. While formal double-coding was not performed, selected transcripts and emerging themes were discussed iteratively with the wider research team (PL, HM-A), including during structured analytical meetings. This collaborative approach supported reflexivity and strengthened the trustworthiness of theme development through peer debriefing and triangulation. The theme and subthemes were then developed by GS and discussed with MM, CR, PL and HM-A. This process later helped structure themes within the preparedness cycle framework, with careful avoidance of preconceptualised notions. ChatGPT (OpenAI, 2024, San Francisco, California, USA) was used to generate alternative phrasings for the labels used in theme titles, which were evaluated and revised by the authors.

Participants did not provide feedback on the findings for the scientific research publication, although a final debriefing to discuss them is planned. Grammarly (Grammarly, 2024, San Francisco, California, USA) was used to improve grammar and style throughout the manuscript.

### Research team reflexivity

The research team for this study consisted of multiple individuals who contributed to various aspects of the research design, data analysis and interpretation. GS is a female medical doctor and researcher from Italy, with previous experience in qualitative research. She is also a climate change and human rights activist. GS contributed to the study design, coordinated the activity planning, performed the interviews, analysed the data and drafted the manuscript. Conducting the research in Italian helped minimise interpretation errors. Her background in local health districts and as a public health medical resident enabled her to build rapport with interviewees, fostering mutual understanding and reducing bias based on participants’ response proficiency. It is acknowledged that key stakeholders may have had an interest in showcasing their response to the floods.

PL, HM and JVS, three senior Swedish researchers, contributed to the study design and the content analysis discussions and provided feedback on the manuscript draft. Being international researchers, they did not have direct working contacts with the Italian health authority where the study was based and could therefore approach content from an external perspective.

MM liaised with Italian authorities and, with CR, participated in the pilot study and content analysis feedback. Although MM and CR worked in the local health authority, they were not involved in the floods and heatwave response. Their expertise contextualised the data interpretation.

## Results

Among the 11 targeted participants, one approached the mayor’s office representative did not reply and could not be included in the study. To preserve anonymity, participant roles and locations were generalised where possible (table 1).

Significant topics such as the identification of vulnerable individuals or planning protocols were analysed for similarities and differences across various dimensions: (1) comparing heatwaves to floods, (2) contrasting perspectives of high-level managers with those of lower-level coordinators and (3) evaluating perspectives from individuals within the health system against those from other institutions.

We identified an overarching theme ‘flexible collaboration: adapting together in crisis’ supported by three themes, as seen in table 2.

**Table 2:** Themes

Overarching theme	Theme	Subthemes
**Flexible collaboration: adapting together in crisis**	**Multilevel collaboration** fosters **adaptability** in the implementation of the plan	**Subsidiarity versus hierarchy**: a balance of coordinated powers for a timely and locally-led response
		**Established routines and community partnerships** are entry points to adaptation strategies
		An **inclusive revision process** assures closure and improvement
	Prioritisation of **consistency, high engagement and flexibility** are key to responsiveness	**Practising the plans** is key to creating cohesiveness, but is often not a priority
		**Synergy and local adaptability** drive healthcare services' responsiveness
	**Rethinking vulnerabilities** and **risk perceptions** in Climate Crises	Differentiation of **risk groups** by type of crisis must be explored
		Limitations in **grasping the impact** of invisible threats

### Theme 1: multilevel collaboration fosters adaptability in the implementation of the plan

Participants emphasised the importance of multilevel collaboration between governance levels and stakeholders (eg, healthcare, police, volunteers) to effectively respond to crises. This collaboration allowed for learning from past experiences, updating strategies and navigating uncertainties.

A reported point of strength was the local volunteer’s involvement, who plays a crucial role in Italy’s emergency system and whose involvement is shaped by regional and cultural contexts. Maintaining strong relationships with these volunteers and local communities was essential for effective and contextually relevant actions.

“In Emilia Romagna, volunteering is very structured. It is a truly important treasure that we have here” (health district management)

On the other hand, the flood crisis created significant top-down communication challenges at times. As a result, several participants suggested that a debriefing and revising moment would be a good opportunity to reflect on improvements that incorporate the observations of responders at all levels further contributing to recovery and adaptation. This could also contribute to closure after such traumatic events.

“A moment of debriefing, in which we tell ourselves what the strengths and weaknesses were and what if it were to happen again. […] However, going back to normal requires already so much energy and you are then immersed in your everyday life.” (nursing services management)

We further grouped the impact of multilevel collaboration on preparedness and response as understood by the participants into three main subthemes.

### Subsidiarity versus hierarchy: a balance of coordinated powers for a timely and locally led response

Clearly defined roles within hierarchical structures were essential for effective crisis response, particularly in overwhelming situations like floods. The subsidiarity principle emphasised local-level action, ensuring that decision-making and problem-solving occurred closer to affected communities, with municipalities serving as the first line of defence.

“This step gave us, as the local health authority, the possibility of maintaining clear coordination of resources and daily feedback…allowing us to address emotional, psychological and social needs through integrated interventions with social services” (psychological service management)

This approach was reflected in practical actions, such as identifying appropriate points of contact across institutions based on the needs of the population.

“If the citizen perhaps needed a bag of sand because they had a house that could soon flood, the municipality took care of it, but when, for example, there was a need to activate a helicopter that was necessary to go to help isolated families, the mayor did not have the direct possibility of activating such a resource and that goes to the provincial and regional level.” (prefecture office)

### Established routines and community partnerships are entry points to adaptation strategies

Participants noted that local healthcare systems and social services have integrated climate-related crisis management into routine practices, with volunteer associations playing a key role in supporting elderly populations (table 3). Prevention measures, such as media campaigns and direct outreach, are coordinated at the municipal level to provide personalised advice to vulnerable individuals.

In contrast to heatwaves that occur more frequently, established routines for responding to floods were less well-defined and discussed.

**Table 3:** Local heatwave procedures

Period	15 May to 15 September
Activation	Regional directive
Components	Detailed weather forecast (based on discomfort index)
	Vulnerable people identification (by general practitioners, social workers, primary healthcare workers, families, clinics for chronic disease management, etc)
	Information and awareness campaigns
	Early warning systems to alert healthcare facilities, vulnerable populations and responders
	Active surveillance during heatwaves,
	Media campaigns (implemented with a specific focus on vulnerable and susceptible people by primary healthcare workers and social workers)

Existing approaches were generally perceived as well-functioning, focusing on chronic condition management and healthy lifestyle promotion. However, there was some recognition of heatwaves as an evolving and changeable threat and some participants expressed doubt about the effectiveness of current strategies, pointing out a lack of critical evaluation and monitoring.

“What is done is a bit of a basic default job, with an effectiveness that I don't know what it is” (mayor’s office)

### An inclusive revision process assures closure and improvement

Responders expressed fatigue and frustration from handling multiple crises (COVID-19, floods, earthquakes) and emphasised the need for inclusive revision processes to ensure resilience.

“The rescue system works. But it’s a shame, […] to have a country that’s […] so vulnerable to all these emergencies. A lot is done to plan, to prevent and to prepare. But then event after event takes place. We need to create a preparedness culture.” (prefecture’s office)

An inclusive revision process could have also picked up on lessons learnt from differences in response to different crises. Participants gave examples of how lessons learnt from one crisis, such as floods, were applied quickly in subsequent emergencies, that is, earthquakes, demonstrating the value of continual adaptation. They reported that during the floods, it quickly became obvious that the integration of psychological support and the family nurse as a community reference point was key to managing the response and assessing needs. These multiple shocks have highlighted the need for emotional and psychological support, which some participants thought should be integrated as part of the crisis unit response.

Participants noted, however, that lessons learnt from one crisis, like COVID-19, floods and earthquakes, cannot always be directly applied to others. Heatwaves were, for example, not perceived as an unusual and singular crisis but rather as a seasonally occurring and normalised threat. Participants said this offers an opportunity for a revision process to update and integrate new lessons from previous iterative heatwaves. They believed municipalities should also consider new administrative frameworks and tools to effectively respond to heatwaves, which may be more complex than other emergencies because they are insidious, underappreciated and affect larger and less well-defined areas.

“It must be an approach that is also regulatory [on a political and administrative level] because I don’t think we can order, for example, a lockdown due to the heat.” (mayor’s office)

### Theme 2: prioritisation of consistency, high engagement and flexibility is key to responsiveness

This theme raises some of the important characteristics of successful responsiveness acknowledged by participants that also rely on flexible collaboration. Participants recognised the importance of a cohesive and synergistic approach by public services in prioritising and addressing timely community needs during crises. As part of that, consistency—intended as behaving or performing always in a similar way—was easier to achieve when the same individuals who managed these roles during normal times also handled them in emergencies. This fosters long-term consistency, reduces role redundancy, ensures accountability and facilitates timely responses.

“The trick is to identify the person who does that thing in peacetime and the same thing must be done in an emergency, it should not be another person […] I have to know how to do that same thing in an emergency, in a tight time frame […] I can't improvise in an emergency.” (civil protection office)

In responding to climate disasters, like floods and heatwaves, participants recognised the value of continuously learning from past experiences as well as making use of the flexibility and adaptability of plans to be able to refine strategies to enhance preparedness and resilience in the present and going forward.

“There are plans which, no matter how detailed they try to be, never enter the operational specifics, which are affected both by the place and the context and by how it occurs.” (nursing services management)

These plans shouldn't be rigid one-size-fits-all scripts, but frameworks that outline roles and responsibilities across functions (public health and veterinary, volunteering, essential services, etc), particularly for the diverse challenges posed by climate-related events.

“This is a job that must be done with a solid basis, but not with a rigid scheme because then the emergency puts you face to face with things that cannot be foreseen.” (mayor’s office)

We can further develop the reflections into three subthemes perceived to additionally contribute to successful responsiveness through prioritisation of consistency, high engagement and flexibility.

### Practising the plans is key to creating cohesiveness, but it is often not a priority.

As part of the importance of maintaining consistency in successful responsiveness, participants reinforced the concept that effective crisis management relies on routinely updating the plan and conducting regular exercises to foster cohesiveness and preparedness. Cohesiveness was intended as cooperation, collaboration and mutual support to reach a shared goal. Participants reported that multidisciplinary exercises were crucial in identifying overlooked aspects and supporting structural improvements and corrective measures. But competing priorities often push training to the background.

“Many times, training is lost over the years, when there are no events. You get absorbed by all the other emergencies that happen.” (civil protection office)

On the other hand, during this specific period, the lack of practice was replaced by ‘training in real life’ with several health emergencies following one another (COVID-19, floods, earthquakes). This allowed the response coordination management to be oiled and functional when needed.

“COVID-19 certainly trained us […] although with different levels of intensity, the mechanism was always running” (health district management)

### Synergy and local adaptability drive healthcare services’ responsiveness

Participants highlighted the synergy between stakeholders (government, businesses, non-governmental organisations (NGOs), volunteers) in ensuring timely, coordinated responses. This was facilitated by the early warning system, which activated timely, coordinated actions to prioritise vulnerable populations and essential services, such as maintaining healthcare access and supporting high-risk facilities.

“We had information about what was about to happen and we immediately began to define operational plans, so we convened what is called our crisis unit, with the heads of the emergency department and services, and the operating theatre coordinator, precisely trying to supervise those areas where we cannot afford to weaken the staff, and therefore we have also begun to pre-alert the staff.” (nursing services management)

Participants also explained how healthcare services leveraged existing community and volunteer partnerships and networks to tailor response strategies to local contexts and needs.

For example, in areas where pharmacies were flooded, the supply of medicines needed by the population of that area was managed by other pharmacies in the local network or by setting up temporary clinics when doctors’ offices were inaccessible to be able to stay close to their patients. It showcased the adaptability and responsiveness of healthcare services in tailoring their approach based on the nature of the emergency and the short-term needs of the affected territory.

“In this case, we entered the territory and, together with the civil protection, the local authorities and the community agents most directly involved, we tried to identify a method of intervention which was different depending on the type of emergency and territory we are going to consider. We have arranged, for example, a primary care medical presence in addition to what was the primary care of general practitioners for a certain time slot of the day, for a couple of weeks, in the most affected areas.” (primary healthcare management)

In summary, participants highlighted the exceptional level of flexibility and engagement across different domains and levels that enabled effective healthcare responses during crises.

“We were all on the field, basically.” (prefecture’s office*)*

### Theme 3: rethinking vulnerabilities and risk perceptions in climate crises

As part of the flexible collaboration necessary for successfully adapting to a crisis, participants pointed to the importance of understanding the possibility of shifting vulnerability across groups depending on the specific crisis as well as challenges of risk perceptions that are incongruous with the imminent threat. This theme can be summarised into two subthemes.

### Differentiation of risk groups by type of crisis must be explored

Participants expressed dissatisfaction with the current mapping of vulnerable populations which is overly reliant on a generic approach for all hazards. They highlighted the need for agile and adaptable strategies informed by better quality data collection, updated IT technologies and data sharing. This would enhance not only emergency responses but also routine healthcare.

“This doesn’t just concern the emergency, it concerns everything else that concerns healthcare, the prevention of flare-ups of chronic pathologies and so on. We have many channels (for identifying vulnerable individuals), but many times they are not so well integrated. So, I think we can do more.” (primary healthcare management)

During the alert period and the following floods, participants said the threshold for identifying vulnerability shifted from traditional criteria that typically included the elderly and those with chronic conditions, to considering new criteria encompassing rapid assistance for groups like children, psychiatric patients and those needing life-saving treatments.

“Even families with complex situations, perhaps with some problems bordering on slightly disadvantaged mental health, not known [by the healthcare system]: that is a whole part of the needs that emerged in that reality. We did not know them before, we had to manage those on the spot, and I must say that a system that allows you to have all these functions around the same table is very operational [in doing that]”. (health district management)

Unexpectedly, even business owners needed psychological support after their facilities were destroyed, emphasising how the community’s social and psychological needs often outweighed clinical considerations in defining vulnerability.

“It wasn’t so much just organic physical problems, but it was: not sleeping at night, the flashbacks, the reactivity, the anguish, the crying.[…] We had children who no longer slept, they wanted to stay awake, alert and dressed because they were terrified that things would happen at night, and they were unprepared to escape.” (psychological health management)

For heatwaves, instead, despite the reported scarcity of reflections and evaluations on the matter, participants expressed the need to rethink vulnerabilities beyond elderly populations, including outdoor workers in agriculture and construction, who face systemic inequalities and exploitation.

“If you have to harvest that day, you harvest that day and therefore there is a real issue that mixes the type of work that is adopted in agriculture with the rules or the non-rules that might or not exist or be followed in agriculture, in gangmastering [ie, workers being illegally employed in the agricultural sector. at very low wages], in the extreme exploitation of work, especially of foreign people.” (mayor’s office)

### Limitations in grasping the impact of invisible threats

Participants reported difficulties in understanding the impact of less visible threats like heatwaves, which were often perceived as less urgent compared with more visible dangers like floods.

“I don’t see heatwaves being perceived by many as a real danger. Indeed, there is a difference which is that when there is a flood or there is a [concrete] danger, even with this lack of understanding, there is nevertheless a somewhat general alarm.” (mayor’s office)

Separately for floods, the lack of prior experience made it harder for people to grasp the severity of the situation, reducing proactive responses.

“They [citizens] told me: “I had water, it had already reached my ankles in the house, but I didn’t believe it could rise more because it had never happened before. I have been living in this house for 70 years; it has never happened”. The human mind tends to say, “If it has never happened, it won’t happen.” (mayor’s office)

## Discussion

**Figure 2:**
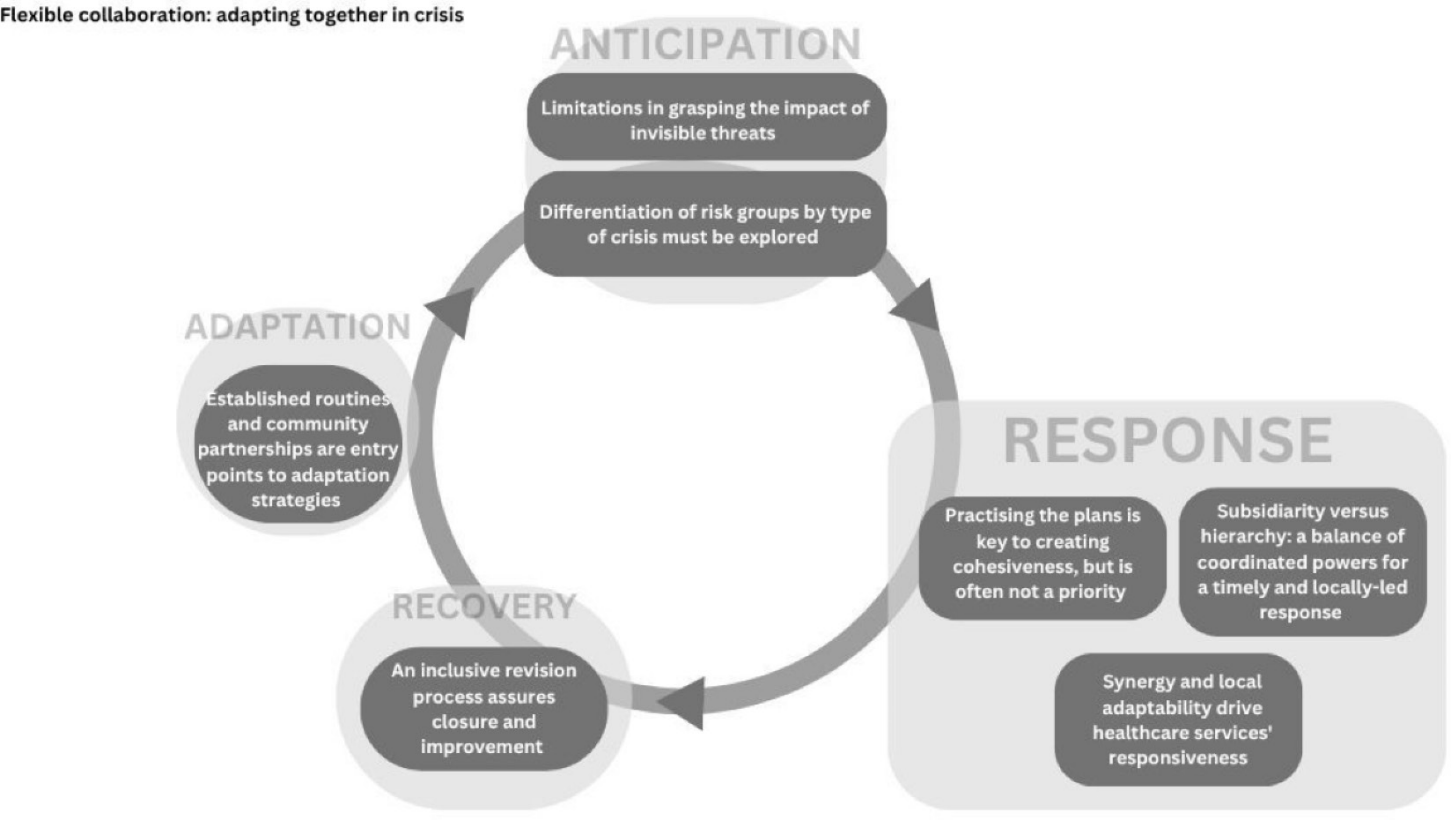
The preparedness cycle in relation to the main subthemes.

This study examines strategies for building climate adaptation and response capacity to the most vulnerable healthcare needs through public health emergency preparedness in Emilia-Romagna, Italy, focusing on floods and heatwaves. Using the preparedness cycle framework, with an added ‘adaptation’ phase, our findings underscore the need for continuous preparedness cycles that integrate lessons learnt for better future response. Each subtheme within this framework—ranging from collaboration, subsidiarity and evolving vulnerabilities—is essential to ensuring flexible, community-based responses that strengthen resilience over time (figure 2).

### Flexible collaboration and subsidiarity

The study reveals that flexibility and multilevel collaboration are essential for the region’s climate emergency response, as highlighted in other studies.^43 44^ Stakeholders emphasised that coordination across different levels of governance—healthcare, emergency services, local authorities and volunteer organisations—fosters adaptability. Subsidiarity ensured decision-making occurred close to affected communities, with municipalities acting as the first line of defence during floods and escalating issues to higher authorities when needed. This decentralised approach ensured that response efforts were timely, locally led and adapted to community-specific conditions.

Our findings indicate that coordination primarily focuses on response, with less integration of community engagement in long-term recovery planning. Enhancing recovery strategies by involving local actors remains a challenge.

### Strengthening coordination mechanisms and long-term resilience

The findings also highlight that effective coordination through community partnerships and volunteer networks is crucial for mobilising healthcare services, yet the system remains reactive rather than proactive.^45^ In contrast, studies in Pakistan and the UK reveal how lack of collaboration, synergy and preparedness can critically undermine response efforts.^46 47^ Simulations, debriefings and collaborative learning post crisis are underused yet vital for solidifying lessons learnt and enhancing future preparedness.^48^ Routine evaluations and an inclusive review process would help mitigate responder fatigue, ensure knowledge transfer and refine strategies based on real-time experiences. These and other recent floods in Italy demonstrate that without consistent preparedness cycles, the healthcare system risks being overwhelmed, especially when multiple crises overlap.^49 50^

### Learning by doing and skills transferring

Interestingly, many participants credited their ability to manage floods to lessons learnt during the COVID-19 pandemic, which streamlined decision-making processes and built confidence in handling large-scale emergencies.

New tools like psychological support and the family nurse, first implemented during the floods, indicate a shift towards more community-based healthcare models. This ability to transfer skills, strategies and relationships adapting to different crises is key to coping with emergent challenges, reflecting the system’s capacity for adaptation, yet they also highlight the need for more proactive planning rather than reactive responses.^48 51^ Italy’s civil protection plans emphasise this adaptability by employing an all-hazards approach.^52^

### Evolving vulnerabilities and inclusive risk perception

Our study also revealed that existing vulnerability definitions, while useful, are insufficient to address the complexities of climate crises. Participants emphasised updating vulnerability mapping to include new groups, like outdoor workers or people with disabilities, who emerged as vulnerable in floods.^53^ This dynamic nature of vulnerability suggests that fixed definitions cannot fully capture the changing realities on the ground, as already shown by previous studies.^54^

Social and psychological factors also play a crucial role in how people experience and respond to climate events. Participants emphasised that addressing vulnerabilities should go beyond clinical needs to include a broader social context. The need for holistic approaches, combining medical, social and psychological support, was echoed across all participants, who were stressing the importance of tailored interventions that adapt to the community’s evolving needs, as highlighted by studies in Nigeria, the UK and India.^55 56^

Furthermore, risk perception remains a challenge, particularly for invisible threats like heatwaves, highlighting the need for targeted public awareness campaigns.^57^,^61^ The recurring nature of heatwaves requires updated strategies, as their frequency, duration and impacts continue to evolve, posing new challenges to public health systems that rely on outdated models of preparedness.

## Strengths and limitations

Conducted soon after significant events, the study provides timely and contextually relevant findings on local preparedness and resilience. The purposive inclusion of key institutional decision-makers—those directly responsible for preparedness and response functions—allowed for indepth exploration of system-level coordination. Additionally, triangulation of interview findings with a document review of relevant preparedness plans enhanced the credibility and analytical depth of the study.

While the sample size was relatively small (n=10), this is consistent with qualitative research standards focused on depth over breadth. Sampling was guided by the concept of information power, ensuring that participants were selected based on their direct relevance to the study objectives. Data saturation was considered reached when no new codes or themes emerged in the final interviews, a judgement made collaboratively within the research team.

Nonetheless, several limitations should be acknowledged. The exclusive focus on high-level responders omits insights from lower-level staff and community members, though this helped maintain a clear focus. Potential participant bias exists, but the candid nature of interviews minimised this concern. Finally, although conducting the study in Italian may have minimised misinterpretations during data collection, translating the findings for an international audience could introduce biases or result in the loss of critical nuances for the analysis.

## Conclusion

Our study aligns with broader frameworks that emphasise the iterative nature of preparedness, where cycles of anticipation, response and recovery are continuously refined. This study provides suggestions that flexible collaboration, effective communication and continuous adaptation are essential for enhancing healthcare preparedness for vulnerable populations in the face of climate-driven extreme weather events. Specifically, our findings suggest that: (a) multilevel collaboration among responders can significantly improve crisis management, as highlighted by participant insights; (b) adapting preparedness strategies to incorporate psychological support reflects a growing understanding of the holistic needs of communities during emergencies; (c) updating vulnerability definitions to include diverse populations ensures that healthcare systems can respond effectively to evolving threats.

While most themes mapped to established preparedness phases, we identified one theme: ‘established routines and community partnerships are entry points to adaptation strategies’—that illustrates how prior experiences and community-based practices can support the integration of adaptation into long-term planning. This supports the inclusion of an ‘adaptation’ step in the preparedness cycle.

Thus, the findings underscore the necessity for systemic changes in preparedness frameworks to adequately address evolving vulnerabilities. Future research should explore the implementation of these strategies across different regions to validate their effectiveness in enhancing resilience.

## Supplementary material

10.1136/bmjph-2024-002459 online supplemental file 1

10.1136/bmjph-2024-002459 online supplemental file 2

## Data Availability

All data relevant to the study are included in the article or uploaded as supplementary information.
