# Supplementary figures and images for: Responder perspectives on preparedness for healthcare needs of vulnerable populations during floods and heatwaves: a qualitative study in Emilia-Romagna, Italy
Source: BMJ Public Health. 2025 Sep 12;3(2):e002459. doi: 10.1136/bmjph-2024-002459 (PMC12519328; doi:10.1136/bmjph-2024-002459)

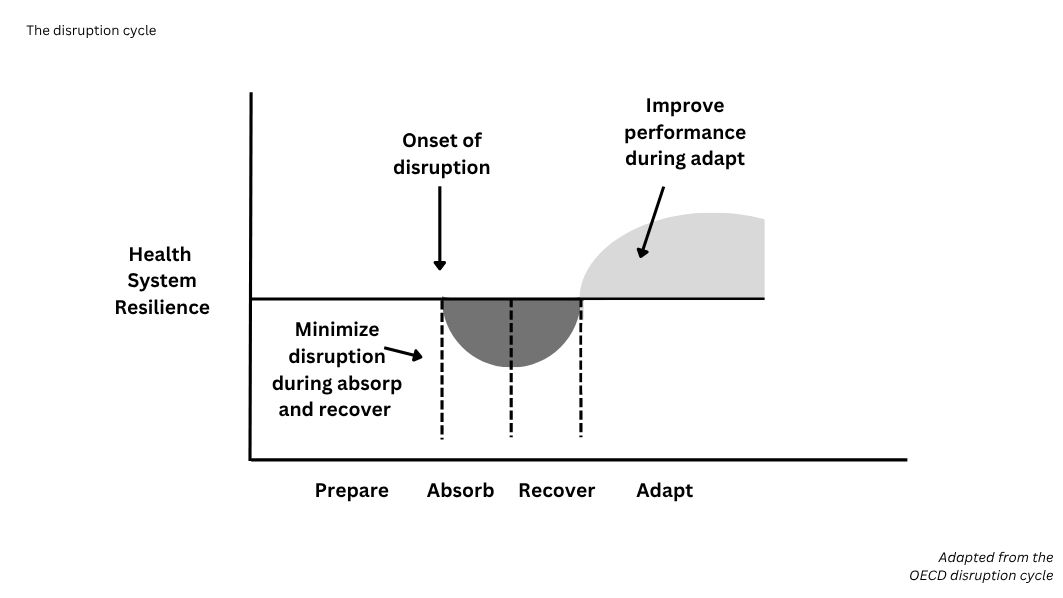

Supplement: online supplemental file 1 [file bmjph-3-2-s001.jpg]
