# Supplementary material for: Responder perspectives on preparedness for healthcare needs of vulnerable populations during floods and heatwaves: a qualitative study in Emilia-Romagna, Italy
Source: BMJ Public Health. 2025 Sep 12;3(2):e002459. doi: 10.1136/bmjph-2024-002459 (PMC12519328; doi:10.1136/bmjph-2024-002459)
Supplement: online supplemental file 2 [file bmjph-3-2-s002.docx]

**Supplementary material**

**Supplementary Figure 1. The disruption cycle**

**Supplementary Table 1. Documentation analysed**

| **Document Title** | **Source Type** | **Geographical coverage** | **Description/Content** | **Date** |
| --- | --- | --- | --- | --- |
| Intermunicipal Civil Protection Plan - Union of Valle del Savio Municipalities | Report, Action Plan | Local | Introduction and Purpose, Context and Risk Analysis, Organizational Structure, Standard Operating Procedures, Available Resources, Communication and Information, Training and Exercises, Coordination with Foreign Entities, Evaluation and Review | 2020 |
| National Prevention Plan  of the effects of heat on health | Report, Action Plan Guidelines | National | Summary of scientific evidence, National Operation Plan, Air Pollution prevention effects | 2019 |
| Survey and monitoring sheet for the Cesena District  Social and health organization data on mitigation of the impact of any heat waves - SUMMER 2023 | Technical file | Local | Territorial Coordination (roles, functions, contacts), Operational Group (roles, functions, contacts), Map of subjects at risk (indicators), Action Plan, Involvement of local community, Informative Actions (targets and actions) | 2023 |
| Survey and monitoring sheet for the Forlì District  Social and health organization data on mitigation of the impact of any heat waves - SUMMER 2023 | Technical file | Local | Territorial Coordination (roles, functions, contacts), Operational Group (roles, functions, contacts), Map of subjects at risk (indicators), Action Plan, Involvement of local community, Informative Actions (targets and actions) | 2023 |
| Survey and monitoring sheet for the Rubicone District  Social and health organization data on mitigation of the impact of any heat waves - SUMMER 2023 | Technical file | Local | Territorial Coordination (roles, functions, contacts), Operational Group (roles, functions, contacts), Map of subjects at risk (indicators), Action Plan, Involvement of local community, Informative Actions (targets and actions) | 2023 |

**Interview Guide (English translation)**

Good [morning/afternoon/evening]. Thanks for being here today. My name is Giorgia Soldà and with the Karolinska Institutet in Sweden and the University of Bologna I am conducting a qualitative research study on preparedness for climate emergencies from a public health point of view. The aim of this study is to explore and gain a deeper understanding of how the health needs of vulnerable people are addressed in these situations.

Today we will conduct a semi-structured interview as part of this research process. Semi-structured interviews provide the opportunity to share thoughts, experiences and perspectives on the topic under investigation. This means that while there are some basic questions I will ask you to answer, there will also be space for you to elaborate, provide examples, or add other elements that you think are important.

Your participation in this study is entirely voluntary and your responses will be kept confidential. We encourage you to express your thoughts openly and honestly.

The interview will be recorded to facilitate the transcription and analysis process; in fact, we will transcribe it in full to analyze its content later. Feel free to tell me at any time if you feel uncomfortable taking notes.

Also, remember that there are no right or wrong answers. They are interested in hearing your unique perspectives and experiences, so don't hesitate to share your thoughts openly. Your participation in this study is greatly appreciated and your contribution will be critical to advancing our knowledge in this area.

Before we get started, if you have any questions or concerns, don't hesitate to ask.

*Identification*

First name

Workplace

Role during the May 2023 flood emergency and the 2023 heat wave response

*Warm-up*

How long have you been working in these positions?

How did you experience these events (free narration)?

Let's start with a timeline to help us.

The floods began at the beginning of May in Emilia-Romagna and the most catastrophic days in Romagna and in the province of Cesena-Forlì were those of 16-17 May, bla bla

With the next questions, we want to focus on managing vulnerable population groups to understand how they are identified and managed during weather warnings.

*Core questions*

1. *Based on your experience, how do you think vulnerable groups should be defined?

2. *Which groups would you include from a socio-health point of view?

3. *Who do you think should be responsible for identifying vulnerable groups?

4. *What health needs should be included and how would you select them in your opinion?

I would like to ask you the same questions regarding heat waves. From what I understand we have had three major heat waves this summer in Italy (second half of June, about half of July, first half of August), and in general the heat wave management period goes from May 15th to September 15th, correct?

5. How would you assess risks to vulnerable populations?

6. What other stakeholders do you think should be involved in managing the health needs of vulnerable populations before and during these emergencies? How can vulnerable populations and their health needs be tracked/recorded?

*Responsibilities and development of preparedness plans:*

7. How and by whom are the plans drawn up? Who is involved in the healthcare sector?

8. How is the health system involved in these plans and what are the roles and responsibilities?

9. How are the capabilities and capabilities identified through the plans built?

10. How are these plans tested?

*Management/actions of preparedness and response plans:*

11. Which stakeholders are involved in health management during the response and with what roles?

12. Who coordinates actions directed at vulnerable populations once the alarms have been raised (yellow, orange and red alerts)? Do you think this system works? Who is responsible for addressing/taking care of their healthcare needs?

13. What actions are taken by health services and stakeholders after receiving alerts from early warning systems for floods and heatwaves?

14. Who is responsible for deciding what actions to take following different alert levels? How were these actions planned?

*Response evaluation and plan reviews:*

15. How are these actions monitored and who is responsible for them??

16. Is there an evaluation process in place to see what worked and what didn't in addressing vulnerable groups during an emergency? How does it work?

17. Are these plans reviewed based on the above assessment? As?

18. How have preparedness plans facilitated the management of the crisis, especially in terms of ensuring the health needs of vulnerable groups?

19. Given the past crisis, what do you think should be included or developed in preparedness plans to improve the management of the health needs of vulnerable groups?
